# Prevalence of retinal nerve fiber layer defects: The Korea National Health and Nutrition Examination Survey 2008–2012

**DOI:** 10.1371/journal.pone.0186032

**Published:** 2017-10-05

**Authors:** Kyeong Ik Na, Jin Wook Jeoung, Won June Lee, Young Kook Kim, Chan Yun Kim, Ki Ho Park, for the Epidemiologic Survey Committee of the Korean Ophthalmologic Society

**Affiliations:** 1 Department of Ophthalmology, Kangdong Sacred Heart Hospital, Hallym University College of Medicine, Seoul, Korea; 2 Department of Ophthalmology, Seoul National University Hospital, Seoul National University College of Medicine, Seoul, Korea; 3 Department of Ophthalmology, Institute of Vision Research, Yonsei University College of Medicine, Seoul, South Korea; Universidade Federal do Rio de Janeiro, BRAZIL

## Abstract

**Purpose:**

To investigate the prevalence and associated factors of retinal nerve fiber layer defects (RNFLDs) in a Korean population.

**Methods:**

The Korea National Health and Nutrition Examination Survey (KNHANES) is a population-based study using a complex, stratified, multistage, probability-cluster survey. We performed a review of 2008–2012 data from the KNHANES. Two masked ophthalmologists evaluated fundus photography to detect RNFLDs. All participants underwent ocular and systemic evaluations.

**Results:**

A total of 28,637 subjects aged ≥ 19 years with at least one evaluable fundus photograph were enrolled. The estimated prevalence of RNFLDs in this Korean population was 4.8% (95% confidence interval, 4.4%–5.3%). The estimated percentages of glaucomatous RNFLD and non-glaucomatous RNFLD subjects were 34.4% and 65.6%, respectively. In a multivariate analysis, the prevalence of RNFLDs was significantly associated with older age (*P* < 0.001), male gender (*P* = 0.047), glycosylated hemoglobin (*P* = 0.048), disc hemorrhage (*P* = 0.016), ISNT rule (*P* < 0.001), and vertical cup to disc ratio (*P* < 0.001).

**Conclusions:**

The prevalence of RNFLDs in a large Korean population-based sample with a minimum age of 19 years was 4.8%. RNFLD prevalence was associated with old age, male gender, glycosylated hemoglobin, disc hemorrhage, and glaucomatous optic disc.

## Introduction

Retinal nerve fibers are axon bundles that originate from retinal ganglion cells. They pass through the inner retina and gather to form the optic nerve. In the brain, the optic nerve runs towards and partially crosses at the optic chiasm and forms the optic tract. Most of the nerve fibers terminate in the lateral geniculate body. Retinal nerve fiber layer defects (RNFLDs) can occur due to a number of diseases that damage this pathway. RNFLDs can be found in eyes with glaucomatous optic neuropathy, optic disc drusen, toxoplasmotic retinochoroidal scars, retinal cotton-wool spots, pituitary gland tumors, and as a sequela of optic neuritis due to multiple sclerosis or long-standing papilledema, among other causes.[1]

Since RNFLDs are not present in normal healthy eyes, they are usually considered a pathological finding.[2] Previous studies suggested that RNFLDs play a significant role in the detection of various diseases.[3–8] Since Hoyt et al. reported the implications of RNFLDs in glaucomatous eyes,[9] RNFLDs have become an important tool in the diagnosis of glaucomatous optic neuropathy.[2, 10–13]

The purpose of the present study was to investigate the nationwide prevalence and associated factors of RNFLDs in a Korean population with a minimum age of 19 years. The data analyzed were obtained from the Korean National Health and Nutrition Examination Survey (KNHANES), a large population-based, cross-sectional survey. To the best of our knowledge, this is the first study to report RNFLD prevalence and associated factors in the general Korean population using a 5-year (2008–2012) dataset from KNHANES.

## Materials and methods

### Study population

The KNHANES is a nationwide population-based cross-sectional study conducted by the Ministry of Health and Welfare of the Republic of Korea. The KNHANES uses a stratified multistage cluster sampling method based on National Census Data and involves noninstitutionalized Korean citizens residing in Korea. The data of KNHANES can be considered to represent the entire Korean population.

The Korean Ophthalmological Society has participated in the survey and performed ophthalmic examinations since the latter half of 2008. This study was based on the data of the KNHANES IV-V (2008–2012). The KNHANES IV (2007–2009) selected 4,600 households throughout 200 regions each year, and KNHANES V (2010–2012) selected 3,840 households throughout 192 regions each year. The target populations were 12,528, 12,722, 10,938, 10,589 and 10,069 in 2008, 2009, 2010, 2011 and 2012 respectively. The response rate of participants from 2008 through 2012 was approximately 80.6% on average. In the present study, subjects aged 19 years and older and having an evaluable fundus photograph of at least one eye were enrolled.

This study was performed in accordance with the tenets of the Declaration of Helsinki. As the data of KNHANES are available to the public after removing personal identifiers and being anonymized, the Institutional Review Board of Seoul National University Hospital determined that this study was exempt from requiring their approval.

### Examination

The KNHANES is composed of 3 contents: health interview, health examination, and nutrition survey. The health interview and health examination are performed by interviewers and trained medical staff in the mobile examination centers. The health interview questionnaire varied with age and included questions on medical conditions, education, and economic activities. The health examination consists of anthropometry, blood pressure measurements, blood test, urine test, oral health examination, spirometry, ophthalmic examination, and ear-nose-and-throat examination.

Height and body weight were measured on anthropometry. Waist circumference was measured at the midpoint between the lowest rib and the iliac crest during exhalation. The body mass index (BMI) was calculated as weight (kg) divided by height (m) squared. Systolic blood pressure and diastolic blood pressure were measured on the right arm after the 5-minute stabilization period using a standard mercury sphygmomanometer (Baumanometer; Baum, NY, USA). After three measurements, the average of the second and third blood pressure measurements was recorded. In the blood test, fasting glucose, glycosylated hemoglobin, total cholesterol, HDL-cholesterol, triglyceride, hemoglobin, and hematocrit were analyzed by a central certified laboratory.

Subjects who had a history of treatment with hypertensive medication, or whose measured systolic blood pressure was ≥ 140 mmHg or diastolic blood pressure was ≥ 90 mmHg, were defined as having hypertension. Subjects who had been diagnosed with diabetes by physicians, or had a fasting plasma glucose level ≥ 126 mg/dL, were defined as suffering from diabetes mellitus. Obesity was defined as BMI ≥ 25.

For quality control, the ophthalmic examination was performed by ophthalmologists trained by the Korean Ophthalmological Society National Epidemiologic Survey Committee. Visual acuity based on the LogMAR Scale (Jin’s Vision chart; Seoul, Korea), spherical equivalent using automatic refractometry (KR-8800; Topcon, Tokyo, Japan), intraocular pressure (IOP) by Goldmann applanation tonometry were measured. Slit-lamp biomicroscopy was performed for anterior segment evaluation. Fundus photographs were taken using a non-mydriatic fundus camera with a 45° field angle (TRC-NW6S; Topcon, Tokyo, Japan) in participants aged 19 years and older. Frequency doubling technology (FDT) perimetry with the screening program N30-1 (Humphrey Matrix FDT perimetry; Carl Zeiss Meditec, Inc., Dublin, CA, USA) was also performed.

The detailed method of fundus photography evaluation has been described previously.[14] In brief, according to the KNHANES protocol, two discrete glaucoma reading committees were established, each comprising glaucoma specialists from different institutes. The fundus photographs were evaluated twice, first as preliminary grading, and later as detailed grading. After the preliminary grading, the detailed grading was performed independently by another group of glaucoma specialists who were blind to the participants' other information. Any discrepancy between the preliminary and detailed grading was adjudicated by a third group of glaucoma specialists (K.H.P and C.Y.K.). Cup to disc ratio (C/D ratio), ISNT rule, disc hemorrhage and RNFLD were recorded. ISNT rule was defined as characteristic configuration for disc rim thickness as follows: inferior > superior > nasal > temporal. Disc hemorrhage was defined as a splinter-shaped or flame-shaped hemorrhage at the border of the optic disc. RNFLDs were defined as either localized RNFLDs or diffuse RNFL atrophy. Localized RNFLDs on fundus photography were determined to be present when their width at a 1-disc-diameter distance from the edge of the disc was larger than that of a major retinal vessel, diverging in an arcuate or wedge shape and reaching the edge of the disc. Diffuse RNFL atrophy was defined as a generalized loss of RNFL visibility in the upper or lower retina without localized wedge-shaped RNFL defects, regardless of their width.

Glaucoma was defined according to the criteria of the modified International Society of Geographical and Epidemiological Ophthalmology. Category 1 criteria were applied to subjects with FDT perimetry results comprising a fixation error and false-positive error ≤ 1. Subjects had to show abnormal FDT perimetry results with at least one location of reduced sensitivity compatible with optic disc appearance or RNFLD, and satisfy one of the following criteria: (1) vertical or horizontal C/D ratio ≥ 0.7 or asymmetry of C/D ratio ≥ 0.2 (both values determined ≥ 97.5^th^ percentile for a normal population in KNHANES), or (2) presence of a disc hemorrhage, or (3) presence of RNFLD. Category 2 criteria were applied to subjects with an absence of FDT perimetry results or fixation or false-positive ≥ 1. Subjects had to satisfy one of the following criteria: (1) vertical or horizontal C/D ratio ≥ 0.9 or asymmetry of C/D ratio ≥ 0.3 (both values determined ≥ 99.5^th^ percentile for a normal population in KNHANES), or (2) presence of RNFLD compatible with optic disc appearance. All RNFLDs of the subjects diagnosed with glaucoma were considered glaucomatous RNFLD.

### Statistical analysis

The KNHANES is a stratified multistage clustered probability design. The sampling weights, strata and clusters were included in statistical analysis. The complex sample analysis was performed in accordance with statistical guidelines of Korea Centers for Disease Control and Prevention.

The prevalence of RNFLD and each location were expressed as weight-adjusted estimation of percentages with the 95% confidence interval (CI). The prevalence of RNFLD in each subject was defined as the presence of RNFLD in at least one eye. Differences between RNFLD group and non-RNFLD group were evaluated using generalized linear model for complex samples and chi-square test for complex samples. Univariate and multivariate logistic regression analysis were performed to determine the associated factors of RNFLD. Glaucomatous RNFLD and non-glaucomatous RNFLD groups were compared and analyzed in the same way. In cases where both eyes met the inclusion criteria, the right eye was chosen. Statistical significance was defined as a *P* < 0.05.

## Results

### Prevalence of retinal nerve fiber layer defects

**Fig 1:**
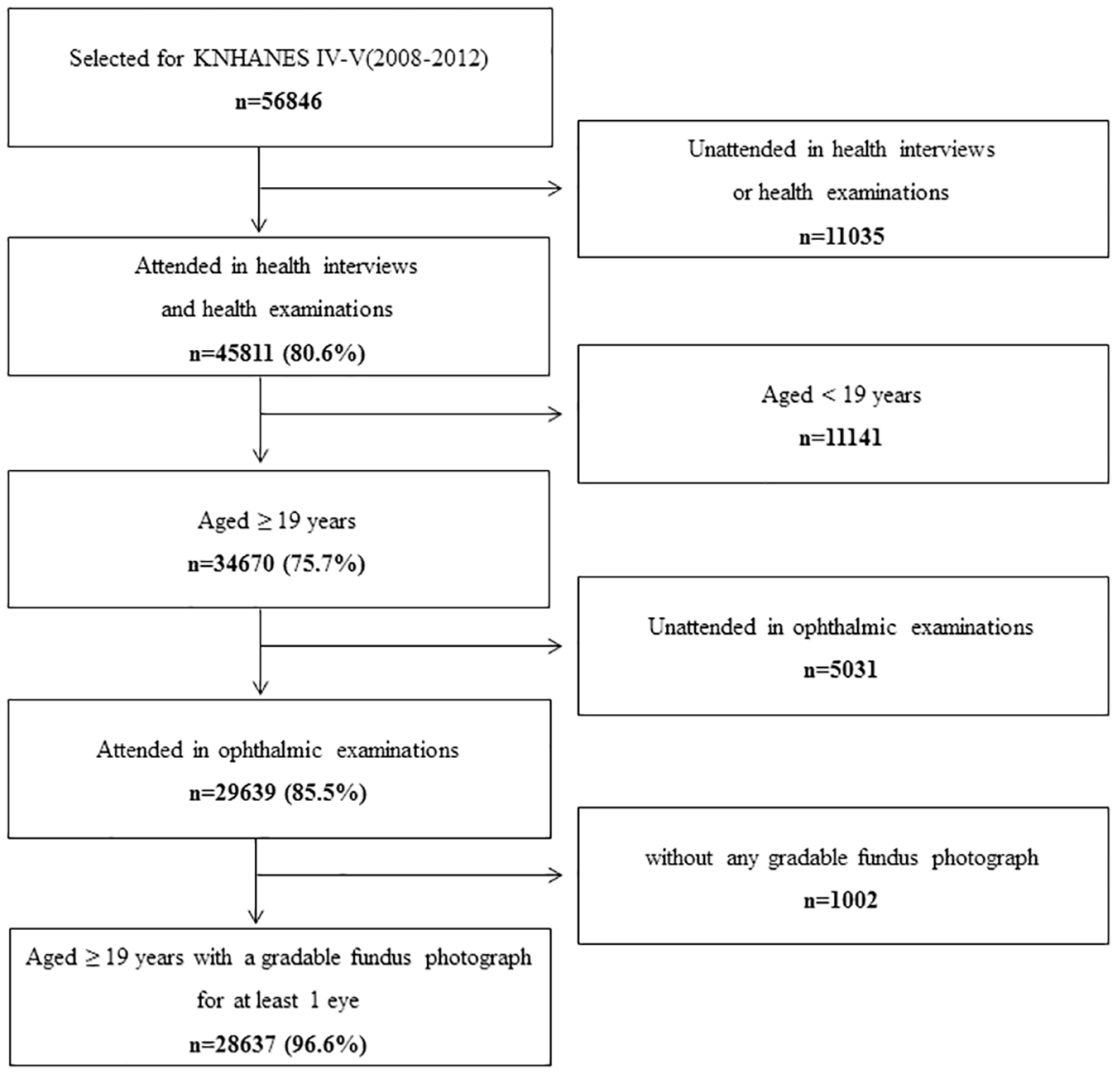
Participation flow chart from the KNHANES 2008–2012.

A total of 56,846 subjects participated in the KNHANES between 2008 and 2012, and 28,637 subjects aged ≥ 19 years with an evaluable fundus photograph from at least one eye were enrolled (Fig 1).

A comparison of the systemic and ocular parameters of the included and excluded participants showed no significant difference between the two groups in the multivariate analysis (S1 and S2 Tables)

Among these 28,637 subjects, 1,474 had a RNFLD in at least one eye. The estimated prevalence of RNFLDs in this Korean population was 4.8% (95% CI, 4.4–5.3). The RNFLD prevalence for the different age groups, 19 to 29 years, 30 to 39 years, 40 to 49 years, 50 to 59 years, 60 to 69 years, and 70 years and older was 1.3%, 3.1%, 4.5%, 7.0%, 8.5%, and 10.4%, respectively. Among 1,474 RNFLD subjects, 553 were diagnosed with glaucoma; the estimated percentages of glaucomatous and non-glaucomatous RNFLD subjects were 34.4% and 65.6%, respectively (Table 1).

**Table 1:** Estimated prevalence of retinal nerve fiber layer defects.

	Prevalence % (95% CI)*	RNFLD subgroups % (95% CI)*	
		Glaucomatous RNFLD	Non-glaucomatous RNFLD
RNFLD	4.8 (4.4–5.3)	34.4 (31.0–37.9)	65.6 (62.1–69.0)
Age group (years)			
19–29	1.3 (1.0–1.9)	22.2 (13.5–34.4)	77.8 (65.6–86.5)
30–39	3.1 (2.5–3.7)	37.8 (28.7–47.9)	62.2 (52.1–71.3)
40–49	4.5 (3.8–5.4)	25.8 (19.6–33.0)	74.2 (67.0–80.4)
50–59	7.0 (6.1–7.9)	31.3 (25.5–37.8)	68.7 (62.2–74.5)
60–69	8.5 (7.4–9.7)	37.4 (31.8–43.3)	62.6 (56.7–68.2)
≥70	10.4 (7.9–13.4)	47.7 (40.9–54.6)	52.3 (45.4–59.1)

RNFLD, retinal nerve fiber layer defect; CI, confidence interval.

*Weight-adjusted estimations of percentage in each group.

Table 2 shows the location of the RNFLDs in 1,474 RNFLD subjects. The unilateral superotemporal region was the most common location followed by the unilateral inferotemporal region. Among a total of 2,348 RNFLDs, 1,357 (57.8%) were located in the superotemporal region and 991 (42.2%) in the inferotemporal region. Among a total of 1,026 glaucomatous RNFLDs, 517 (50.4%) were located in the superotemporal region and 509 (49.6%) in the inferotemporal region.

**Table 2:** Location of retinal nerve fiber layer defects in 1,474 subjects.

RNFLD location	Number of subjects	Prevalence % (95% CI)*	RNFLD subgroups % (95% CI)*	
			Glaucomatous RNFLD	Non-glaucomatous RNFLD
Unilateral ST	602	44.1 (40.7–47.4)	20.0 (15.9–24.8)	80.0 (75.2–84.1)
Bilateral ST	102	7.5 (5.8–9.6)	36.1 (26.3–47.2)	63.9 (52.8–73.7)
Unilateral IT	327	20.5 (18.0–23.3)	41.6 (35.3–48.2)	58.4 (51.8–64.7)
Bilateral IT	66	3.9 (2.8–5.3)	50.1 (38.9–61.4)	49.9 (38.6–61.1)
Unilateral ST and IT	122	8.0 (6.4–10.1)	44.0 (33.4–55.2)	56.0 (44.8–66.6)
ST in one eye and IT in the other eye	38	2.6 (1.8–3.7)	34.6 (22.6–48.9)	65.4 (51.1–77.4)
ST and IT in one eye and ST in the other eye	62	4.7 (3.4–6.5)	50.6 (35.9–65.2)	49.4 (34.8–64.1)
ST and IT in one eye and IT in the other eye	43	2.0 (1.4–2.9)	71.2 (55.7–83.0)	28.8 (17.0–44.3)
Bilateral ST and IT	112	6.7 (5.2–8.8)	61.6 (49.4–72.4)	38.4 (27.6–50.6)

RNFLD, retinal nerve fiber layer defect; CI, confidence interval; ST, superotemporal retinal nerve fiber layer defect; IT, inferotemporal retinal nerve fiber layer defect.

*Weight-adjusted estimations of percentage in each group.

### Risk factors of retinal nerve fiber layer defects

The characteristics of the RNFLD group (1,474 subjects) and non-RNFLD group (27,163 subjects) are listed in Table 3. In the RNFLD group, subjects were significantly older, the proportion of male subjects was higher, and disc hemorrhage and glaucomatous optic disc were more prevalent.

**Table 3:** Systemic and ocular parameters of the retinal nerve fiber layer defect (RNFLD) group and the non-RNFLD group.

	RNFLD	Non-RNFLD	*P*-value	RNFLD subgroups		
				Glaucomatous RNFLD	Non-glaucomatous RNFLD	*P*-value
Age (years)*	53.73	44.63	<0.001‡	56.12	52.47	0.001‡
Male gender (%)†	57.2	49.1	<0.001§	55.2	58.2	0.359§
Height (cm)*	163.08	164.00	0.010‡	162.08	163.61	0.030‡
Weight (kg)*	63.90	63.81	0.836‡	62.54	64.61	0.015‡
Waist circumference (cm)*	83.13	80.81	<0.001‡	82.91	83.24	0.640‡
BMI (kg/m^2^)*	23.92	23.63	0.006‡	23.71	24.03	0.183‡
Obesity (%)†	32.6	31.7	0.573§	30.8	33.6	0.378§
Systolic blood pressure (mmHg)*	125.66	117.64	<0.001‡	126.51	125.21	0.308‡
Diastolic blood pressure (mmHg)*	79.30	76.53	<0.001‡	79.31	79.30	0.988‡
Hypertension (%)†	46.9	26.1	<0.001§	49.4	45.6	0.259§
Fasting glucose (mg/dL)*	103.79	96.26	<0.001‡	102.13	104.65	0.275‡
Glycosylated hemoglobin (%)*	6.12	5.79	<0.001‡	6.22	6.09	0.319‡
Diabetes mellitus (%)†	16.1	7.8	<0.001§	15.6	16.3	0.779§
Total cholesterol (mg/dL)*	187.39	187.30	0.945‡	190.98	185.54	0.036‡
HDL-cholesterol (mg/dL)*	50.58	52.57	<0.001‡	51.14	50.29	0.328‡
Triglyceride (mg/dL)*	149.76	133.25	0.001‡	152.79	148.20	0.683‡
Hemoglobin (g/dL)*	14.24	14.16	0.205‡	14.23	14.24	0.924‡
Hematocrit (%)*	42.18	42.10	0.644‡	42.33	42.10	0.433‡
IOP (mmHg)*	14.2873	13.9472	0.003‡	14.6256	14.1108	0.017‡
Disc hemorrhage (%)†	2.0	0.1	<0.001§	2.9	1.6	0.120§
ISNT rule (%)†	61.1	94.7	<0.001§	21.6	86.2	0.000§
Vertical C/D ratio*	0.5351	0.3555	<0.001‡	0.6651	0.4672	<0.001‡
Horizontal C/D ratio*	0.5069	0.3586	<0.001‡	0.6105	0.4527	<0.001‡
Spherical equivalent (diopter)*	-0.8649	-1.1898	<0.001‡	-1.1635	-0.7090	0.011‡

RNFLD, retinal nerve fiber layer defect; BMI, body mass index; IOP, intraocular pressure; ISNT rule, the thickness of disc rims beginning with the inferior rim followed by the superior rim, the nasal rim, and the temporal rim; C/D ratio, cup to disc ratio.

*Weight-adjusted estimations of numerical values are shown as mean.

^†^Weight-adjusted estimations of percentage in each group.

^‡^Generalized linear model for complex samples.

^§^Chi-square test for complex samples.

In the univariate analysis, the prevalence of RNFLDs was associated with older age (*P* < 0.001), male gender (*P* < 0.001), height (*P* = 0.010), waist circumference (*P* < 0.001), BMI (*P* = 0.005), systolic blood pressure (*P* < 0.001), diastolic blood pressure (*P* < 0.001), hypertension (*P* < 0.001), fasting glucose (*P* < 0.001), glycosylated hemoglobin (*P* < 0.001), diabetes mellitus (*P* < 0.001), HDL-cholesterol (*P* < 0.001), triglyceride (*P* < 0.001), IOP (*P* = 0.002), disc hemorrhage (*P* < 0.001), ISNT rule (*P* < 0.001), vertical C/D ratio (*P* < 0.001), horizontal C/D ratio (*P* < 0.001), and spherical equivalent (*P* < 0.001) (Table 4).

**Table 4:** Univariate regression analysis for systemic and ocular parameters in the retinal nerve fiber layer defect (RNFLD) group, using the non-RNFLD group as a reference.

	Odd ratio (95% CI)	*P*-value
Age (years)	1.034(1.030–1.039)	<0.001
Male gender (%)	1.360(1.224–1.569)	<0.001
Height (cm)	0.990(0.982–0.998)	0.010
Weight (kg)	1.001(0.995–1.006)	0.835
Waist circumference (cm)	1.022(1.016–1.028)	<0.001
BMI (kg/m^2^)	1.023(1.007–1.040)	0.005
Obesity (%)	1.041(0.905–1.198)	0.572
Systolic blood pressure (mmHg)	1.025(1.022–1.028)	<0.001
Diastolic blood pressure (mmHg)	1.023(1.017–1.029)	<0.001
Hypertension (%)	2.497(2.180–2.860)	<0.001
Fasting glucose (mg/dL)	1.010(1.008–1.012)	<0.001
Glycosylated hemoglobin (%)	1.272(1.194–1.355)	<0.001
Diabetes mellitus (%)	2.267(1.858–2.766)	<0.001
Total cholesterol (mg/dL)	1.000(0.998–1.002)	0.945
HDL-cholesterol (mg/dL)	0.987(0.981–0.993)	<0.001
Triglyceride (mg/dL)	1.001(1.001–1.001)	<0.001
Hemoglobin (g/dL)	1.029(0.984–1.076)	0.209
Hematocrit (%)	1.004(0.987–1.021)	0.645
IOP (mmHg)	1.044(1.016–1076)	0.002
Disc hemorrhage (%)	17.123(9.083–32.279)	<0.001
ISNT rule (%)	0.056(0.046–0.068)	<0.001
Vertical C/D ratio	2.011(1.914–2.113)	<0.001
Horizontal C/D ratio	1.846(1.757–1.939)	<0.001
Spherical equivalent (diopter)	1.070(1.032–1.111)	<0.001

CI, confidence interval; BMI, body mass index; IOP, intraocular pressure; ISNT rule, the thickness of disc rims beginning with the inferior rim followed by the superior rim, the nasal rim, and the temporal rim; C/D ratio, cup to disc ratio.

The variables with a *P*-value less than 0.05 after the univariate analysis subsequently were included in the multivariate analysis. Some of the factors were excluded considering the multicollinearity and clinical significance of the factors. The multivariate analysis indicated that RNFLDs were associated with older age (*P* < 0.001), male gender (*P* = 0.047), glycosylated hemoglobin (*P* = 0.048), disc hemorrhage (*P* = 0.016), ISNT rule (*P* < 0.001), and vertical C/D ratio (*P* < 0.001) (Table 5).

**Table 5:** Multivariate regression analysis for systemic and ocular parameters of retinal nerve fiber layer defect (RNFLD) group, using the non-RNFLD group as a reference.

	Odd ratio (95% CI)	*P*-value
Age (years)	1.024(1.015–1.034)	<0.001
Male gender (%)	1.273(1.003–1.615)	0.047
BMI (kg/m2)	1.009(0.974–1.045)	0.604
Hypertension (%)	1.229(0.933–1.619)	0.142
Glycosylated hemoglobin (%)	1.180(1.001–1.390)	0.048
Diabetes mellitus (%)	0.753(0.501–1.132)	0.172
HDL-cholesterol (mg/dL)	1.006(0.995–1.017)	0.279
Triglyceride (mg/dL)	1.000(0.999–1.001)	0.672
IOP (mmHg)	0.977(0.929–1.027)	0.358
Disc hemorrhage (%)	5.252(1.366–20.189)	0.016
ISNT rule (%)	0.290(0.196–0.427)	<0.001
Vertical C/D ratio	1.718(1.563–1.888)	<0.001
Spherical equivalent (diopter)	0.999(0.992–1.005)	0.649

CI, confidence interval; BMI, body mass index; IOP, intraocular pressure; ISNT rule, the thickness of disc rims beginning with the inferior rim followed by the superior rim, the nasal rim, and the temporal rim; C/D ratio, cup to disc ratio.

The characteristics of subjects in the glaucomatous RNFLD group (553 subjects) and non-glaucomatous RNFLD group (921 subjects) are listed in Table 3. After univariate analysis, multivariate analysis was performed in the same way as mentioned above. In the multivariate analysis, glaucomatous RNFLDs were associated with older age (*P* < 0.001), weight (*P* = 0.032), IOP (*P* = 0.022), and spherical equivalent (*P* < 0.001), compared with non-glaucomatous RNFLDs (Tables 6 and 7).

**Table 6:** Univariate regression analysis for systemic and ocular parameters in the glaucomatous retinal nerve fiber layer defect (RNFLD) group, using the non-glaucomatous RNFLD group as a reference.

	Odd ratio (95% CI)	*P*-value
Age (years)	1.017 (1.007–1.026)	0.001
Male gender (%)	0.882 (0.675–1.153)	0.359
Height (cm)	0.985 (0.971–0.999)	0.031
Weight (kg)	0.985 (0.973–0.997)	0.015
Waist circumference (cm)	0.997 (0.982–1.011)	0.639
BMI (kg/m^2^)	0.971 (0.930–1.014)	0.185
Obesity (%)	0.881 (0.663–1.169)	0.379
Systolic blood pressure (mmHg)	1.004 (0.997–1.011)	0.304
Diastolic blood pressure (mmHg)	1.000 (0.987–1.014)	0.988
Hypertension (%)	1.164 (0.894–1.516)	0.259
Fasting glucose (mg/dL)	0.997 (0.992–1.002)	0.281
Glycosylated hemoglobin (%)	1.078 (0.923–1.260)	0.342
Diabetes mellitus (%)	0.948 (0.655–1.374)	0.779
Total cholesterol (mg/dL)	1.004 (1.000–1.008)	0.031
HDL-cholesterol (mg/dL)	1.006 (0.994–1.018)	0.329
Triglyceride (mg/dL)	1.000 (0.999–1.002)	0.649
Hemoglobin (g/dL)	0.996 (0.918–1.080)	0.924
Hematocrit (%)	1.012 (0.982–1.044)	0.434
IOP (mmHg)	1.056 (1.009–1.104)	0.018
Disc hemorrhage (%)	1.818 (0.847–3.903)	0.125
ISNT rule (%)	0.044 (0.030–0.064)	0.000
Vertical C/D ratio	1.901 (1.724–2.097)	0.001
Horizontal C/D ratio	1.619 (1.489–1.761)	0.001
Spherical equivalent (diopter)	0.923 (0.870–0.979)	0.008

CI, confidence interval; BMI, body mass index; IOP, intraocular pressure; ISNT rule, the thickness of disc rims beginning with the inferior rim followed by the superior rim, the nasal rim, and the temporal rim; C/D ratio, cup to disc ratio.

**Table 7:** Multivariate regression analysis for systemic and ocular parameters of glaucomatous retinal nerve fiber layer defect (RNFLD) group, using the non-glaucomatous-RNFLD group as a reference.

	Odd ratio (95% CI)	*P*-value
Age (years)	1.025 (1.012–1.039)	<0.001
Height (cm)	1.018 (0.996–1.041)	0.116
Weight (kg)	0.980 (0.962–0.998)	0.032
Total cholesterol (mg/dL)	1.003 (0.999–1.007)	0.121
IOP (mmHg)	1.061 (1.009–1.116)	0.022
Spherical equivalent (diopter)	0.869 (0.809–0.933)	<0.001

CI, confidence interval; IOP, intraocular pressure.

## Discussion

Previous glaucoma population-based studies have generally analyzed subjects aged 40 years and older,[14–16] potentially due to the relatively old age of subjects with glaucoma. However, RNFLDs can be caused not only by glaucoma but also by other diseases.[1] Non-glaucomatous RNFLDs can occur at a younger age.[17–19] Therefore, the present study investigated the prevalence and associated factors of RNFLDs in subjects aged 19 years and older.

In this study, the estimated prevalence of RNFLDs in a Korean population aged ≥ 19 years was 4.8% (95% CI, 4.4–5.3). In 2012, the total population aged ≥ 19 years in Korea was approximately 40 million, according to resident population statistics. Therefore, approximately 1.92 million Koreans may have RNFLDs. The present findings are similar to those of an earlier study that reported a RNFLD prevalence of 5.4% by examining 4,395 Korean subjects who underwent health checkups.[20] In the Beijing Eye Study,[21] 3,242 subjects were enrolled; the prevalence of localized RNFLD was 14.8% per subject aged ≥ 50 years. When the same age ranges were applied to the present study, in the age groups of 50 to 59 years, 60 to 69 years, and ≥ 70 years, the RNFLD prevalence was 7.0%, 8.5%, and 10.4%, respectively. The discrepancies among these findings may reflect differences in study population characteristics and design among different studies (for example, the Beijing Eye Study^15^ utilized spectral domain optical coherence tomography to detect RNFLDs).

In the present study, the estimated prevalence of glaucoma was 2.6% (95% CI, 2.3–2.8) in a Korean population aged ≥ 19 years and 3.4% (95% CI, 3.1–3.8) in a Korean population aged ≥ 40 years. Previously, Kim et al.[14] reported that the prevalence of primary open-angle glaucoma was 4.7% in a Korean population aged ≥ 40 years using the data of KNHANES (2008–2011). Even if all types of glaucoma were included in our study, such as angle closure glaucoma, the prevalence of glaucoma was still lower than the previous study. Kim et al.[14] evaluated the prevalence of primary open-angle glaucoma using non-glaucoma subjects as a control group and excluded cases not satisfying either of the two following criteria: primary open-angle glaucoma criteria and non-glaucoma criteria. In contrast, we included all the cases from KNHANES, which may have resulted in the lower prevalence of glaucoma in our study.

In the present study, RNFLDs most frequently occurred in the unilateral superotemporal region. The prevalence of unilateral superotemporal RNFLDs was 44.1%, double that of unilateral inferotemporal RNFLDs. The total number of RNFLDs was 2,348, and more RNFLDs were located in the superotemporal region (1,357, 57.8%) than in the inferotemporal region (991, 42.2%). Our results are partly concordant with the investigation by Jeon et al., in which the RNFLDs in patients with diabetes were located predominantly in the superior hemisphere.[22] In patients with glaucoma, previous studies showed that the superior visual field (VF) was more frequently affected than the inferior VF, and the rate of VF loss was more rapid in the superior VF compared with the inferior VF.[23–26] When considering the spatial relationship between RNFLDs and the VF, we can assume that the inferotemporal RNFLDs were more prevalent than superotemporal RNFLDs in glaucomatous subjects. However, among a total of 1,026 glaucomatous RNFLDs, the number of inferotemporal RNFLDs (509, 49.6%) was similar to that of superotemporal RNFLDs (517, 50.4%). As far as we know, there is no comparable population-based study on the location of glaucomatous damage and the prevalence of each location. Further investigations are needed to validate this view and explore other potential explanations that could account for these observations.

In this study, the prevalence of RNFLDs increased with age. The multivariate analysis revealed a significant association between the prevalence of RNFLDs and older age. Previous studies also reported that the prevalence of RNFLDs was significantly higher in older individuals.[21] This can be explained by age-related loss of retinal ganglion cells.[27–29] In the present study, the largest increase (2.5%) in the prevalence of RNFLDs between adjacent age groups occurred between the 40 to 49 years group (4.5%) and the 50 to 59 years group (7.0%). These findings suggest that 50- to 59-year-olds are likely to show the highest incidence of RNFLDs. Further longitudinal studies are needed to elucidate the effects of aging on the incidence rate of RNFLDs.

RNFLDs were associated with glycosylated hemoglobin level in this study. Previous cross-sectional studies have described the association between RNFLD and diabetes mellitus.[7, 30–34] However, in the Beijing Eye Study,[21] there was no statistically significant association between the prevalence of localized RNFLD and glycosylated hemoglobin. In our study, RNFLD was significantly associated with glycosylated hemoglobin level, the only systemic factor, except age and gender, after adjustment for the other factors in the multivariate analysis. This can be explained by the effects of insulin resistance on autoregulatory dysfunction.[35, 36] The dysfunctional vascular autoregulation of the eye produces impaired ocular blood flow, and this phenomenon may cause RNFLD-associated diseases such as glaucoma, retinal cotton-wool spots, and ischemic optic neuropathy.

In the present study, several ocular factors were included in the analysis to evaluate their association with RNFLD. IOP and spherical equivalent were significant factors in the univariate analysis, but not in the multivariate analysis. The IOP is reported worldwide as a risk factor for glaucoma.[37–40] Kim et al.[14] found that a higher IOP was associated significantly with primary open angle glaucoma in a Korean population. However, other diseases presenting RNFLDs were assumed to have weakened the correlation between RNFLD and IOP. Furthermore, in a multivariate analysis, IOP was not significantly correlated with RNFLD.

Disc hemorrhage is an important risk factor for the development and progression of glaucoma.[41–43] Sugiyama et al.[44] reported the occurrence of RNFLDs in 47 (97.9%) of 48 eyes with 64 disc hemorrhages, and the location of 51 (79.7%) disc hemorrhages was shown to coincide with that of RNFLDs. Airaksinen et al.[45] found that the location of RNFLDs could be accurately predicted by the disc hemorrhage. In the present study, the prevalence of disc hemorrhage in the RNFLD group was 2.0% and disc hemorrhage was significantly associated with RNFLD (odd ratio = 5.252 [95% CI, 1.366–20.189]).

Glaucomatous optic disc, defined as increased vertical C/D ratio and against ISNT rule, was significantly associated with RNFLD in the present study. The essential pathologic process of glaucoma is the loss of retinal ganglion cells and their axons. Clinically, loss of retinal ganglion cell axons can be detected as localized RNFLDs or diffuse RNFL atrophy. A previous study reported that RNFL loss is observed in 60% of eyes approximately 6 years before any detectable VF defects.[46] Therefore, evaluation of the RNFL is important in detecting and monitoring glaucoma patients. In the present study, the high percentage of glaucoma subjects in the RNFLD group also suggests a significant relationship between RNFLD and glaucomatous optic neuropathy.

A further analysis was performed to compare the glaucomatous RNFLD and non-glaucomatous RNFLD groups. Glaucomatous RNFLDs were associated with older age, low weight, high IOP, and myopic spherical equivalent, compared with non-glaucomatous RNFLDs. Other known risk factors for glaucoma were not significantly different between the two groups. We supposed that these two RNFLD groups might share common underlying systemic factors affecting RNFLDs. For example, diabetes might be a risk factor for non-glaucomatous RNFLD as well as glaucoma. In contrast, specific local factors affecting the optic nerve head might cause glaucomatous RNFLDs. High IOP[37–40] and myopia[37, 47–49] are well known risk factors of glaucoma. In addition, a previous population-based study reported that low weight tended to be associated with a smaller neuroretinal rim area and larger C/D ratio.[50] Since individual fundus photographs were inaccessible, the exact diagnosis of each non-glaucomatous RNFLD subject is unknown. We speculate that non-glaucomatous RNFLDs are associated with various systemic and ocular conditions, such as diabetes, optic nerve drusen, and retinal cotton wool spots. Further investigations are needed to determine the causes of non-glaucomatous RNFLDs and the frequency of each cause.

This study has several limitations. First, participants with nonevaluable fundus photograph for at least one eye, for any reason, were excluded. This may have therefore introduced selection bias that could have affected our results. Second, since this study was cross-sectional in nature, all parameters were measured and recorded on the examination date only. Therefore, our findings may not reflect potential fluctuations in blood pressure, blood test parameters, and IOP. Despite these limitations, this study is strengthened by the fact that the KNHANES is a nationwide population-based survey of the Korean population, which provides an important foundation for investigating the prevalence and associated factors of RNFLDs in a large data sample.

In conclusion, the prevalence of RNFLDs in a Korean large population-based sample aged ≥ 19 years was 4.8%. The prevalence of RNFLDs was associated with old age, male gender, glycosylated hemoglobin, disc hemorrhage, and glaucomatous optic disc.

## Supporting information

S1 Table Systemic and ocular parameters of study participants and excluded candidates. (DOCX)

S2 Table Multivariate regression analysis for systemic and ocular parameters of excluded candidates, using study participants as a reference. (DOCX)
