# Supplementary material for: Prevalence of retinal nerve fiber layer defects: The Korea National Health and Nutrition Examination Survey 2008–2012
Source: PLoS One. 2017 Oct 5;12(10):e0186032. doi: 10.1371/journal.pone.0186032 (PMC5628941; doi:10.1371/journal.pone.0186032)
Supplement: S1 Table — (DOCX) [file pone.0186032.s001.docx]

**S1 Table. Systemic and ocular parameters of study participants and excluded candidates**

|  | **Included** | **Excluded** | ***P*-value** |
| --- | --- | --- | --- |
| Subjects (n) | 28637 | 1002 |  |
| Age (years)* | 45.07 | 50.88 | <0.001‡ |
| Male gender (%)† | 49.5 | 48.6 | 0.631§ |
| Height (cm)* | 163.96 | 162.29 | <0.001‡ |
| Weight (kg)* | 63.82 | 62.92 | 0.103‡ |
| Waist circumference (cm)* | 80.92 | 81.45 | 0.162‡ |
| BMI (kg/m^2^)* | 23.65 | 23.79 | 0.314‡ |
| Obesity (%)† | 31.8 | 34.2 | 0.189§ |
| Systolic blood pressure (mmHg)* | 118.03 | 122.07 | <0.001‡ |
| Diastolic blood pressure (mmHg)* | 76.66 | 76.91 | 0.623‡ |
| Hypertension (%)† | 27.1 | 37.4 | <0.001§ |
| Fasting glucose (mg/dL)* | 96.62 | 102.54 | <0.001‡ |
| Glycosylated hemoglobin (%)* | 5.81 | 5.95 | 0.033‡ |
| Diabetes mellitus (%)† | 8.2 | 13.3 | <0.001§ |
| Total cholesterol (mg/dL)* | 187.30 | 188.66 | 0.447‡ |
| HDL-cholesterol (mg/dL)* | 52.47 | 52.11 | 0.536‡ |
| Triglyceride (mg/dL)* | 134.04 | 141.15 | 0.127‡ |
| Hemoglobin (g/dL)* | 14.16 | 14.07 | 0.223‡ |
| Hematocrit (%)* | 42.11 | 41.98 | 0.542‡ |
| IOP (mmHg)* | 13.7906 | 13.9708 | 0.240‡ |

BMI, body mass index; IOP, intraocular pressure.

*Weight-adjusted estimations of numerical values are shown as mean.

†Weight-adjusted estimations of percentage in each group.

‡Generalized linear model for complex samples.

§Chi-square test for complex samples.
