# Supplementary material for: Prevalence of retinal nerve fiber layer defects: The Korea National Health and Nutrition Examination Survey 2008–2012
Source: PLoS One. 2017 Oct 5;12(10):e0186032. doi: 10.1371/journal.pone.0186032 (PMC5628941; doi:10.1371/journal.pone.0186032)
Supplement: S2 Table — (DOCX) [file pone.0186032.s002.docx]

**S2 Table. Multivariate regression analysis for systemic and ocular parameters of excluded candidates, using study participants as a reference**

|  | **Odd ratio (95% CI)** | ***P*-value** |
| --- | --- | --- |
| Age (years) | 1.014(0.997-1.030) | 0.104 |
| Height (cm) | 0.994(0.980-1.008) | 0.420 |
| Systolic blood pressure (mmHg) | 0.999(0.989-1.009) | 0.896 |
| Hypertension (%) | 1.074(0.748-1.541) | 0.698 |
| Fasting glucose (mg/dL) | 1.002(0.994-1.010) | 0.686 |
| Glycosylated hemoglobin (%) | 1.067(0.841-1.354) | 0.592 |
| Diabetes mellitus (%) | 0.916(0.620-1.353) | 0.660 |

CI, confidence interval.
